# *In vitro* Release of Eosinophil Proteins in Allergic and Atopic Dermatitis Patients

**DOI:** 10.1155/S0962935194000323

**Published:** 1994

**Authors:** L. K. Poulsen, C. M. Reimert, C. Bindslev-Jensen

**Affiliations:** 1 Laboratory of Medical Allergology Allergy Unit National University Hospital, Medical Department TTA 7542, 20 Tagensuej Copenhagen DK-2200 Denmark; 2 Food Allergy Clinic Allergy Unit National University Hospital, Medical Department TTA 7542, 20 Tagensuej Copenhagen DK-2200 Denmark

## Abstract

To investigate whether eosinophils are stimulated *in vivo* or have acquired an increased susceptibility to stimuli from the coagulation cascade, the release of eosinophil proteins was compared for three groups of donors with different levels of serum IgE. (1) with atopic dermatitis (s-IgE > 5000 IU/ml, *n* = 11); (2) with inhalant allergy (200 < s-IgE < 2 000 IU/ml, *n* = 10); and (3) non-allergic (s- IgE < 100 IU/ml, *n* = 10). The levels of eosinophil cationic protein and eosinophil protein X (ECP, EPX) were determined in serum (clotting time = 2.0 h) and plasma. Serum and plasma ECP in normal donors demonstrated large intra-personal variations (C.V. 50–80%), but serum-ECP (mean 8.1 ng/ml) was clearly distinguishable from plasma ECP (mean 1.0 ng/ml) by a factor of 8 (range: 5.6–11.6). The ECP released during clotting was markedly increased in the atopic dermatitis group (serum:plasma ratio 13.5, p < 0.003) compared with the other groups (6.7 and 5.6). EPX, having a higher plasma level, demonstrated a less pronounced release (serum: plasma ratios 2.0, 1.7 and 1.4), with no statistical difference between donor groups. Considering all donors together the levels of ECP and EPX in plasma and in serum were correlated to the number of eosinophils (coefficients of correlation 0.54-0.58, p < 0.002).


Mediators of Inflammation 3, 223–227 (1994)

### *In vitro* release of eosinophil proteins in allergic and atopic dermatitis patients

L. K. Poulsen,[1,CA] C. M. Reimert[1] and C. Bindslev-Jensen[2]

[1]Laboratory of Medical Allergology and [2]Food Allergy Clinic, Allergy Unit, National University Hospital, Medical Department, TTA 7542, 20 Tagensuej DK-2200 Copenhagen, Denmark

[CA] Corresponding Author

**To investigate whether eosinophils are stimulated *in vivo* or have acquired an increased susceptibility to stimuli from the coagulation cascade, the release of eosinophil proteins was compared for three groups of donors with different levels of serum IgE. (1) with atopic dermatitis (s-IgE > 5 000 IU/ml, *n* = 11); (2) with inhalant allergy (200 < s-IgE < 2 000 IU/ml, *n* = 10); and (3) non-allergic (s-IgE < 100 IU/ml, *n* = 10). The levels of eosinophil cationic protein and eosinophil protein X (ECP, EPX) were determined in serum (clotting time = 2.0 h) and plasma. Serum and plasma ECP in normal donors demonstrated large intra-personal variations (C.V. 50–80%), but serum-ECP (mean 8.1 ng/ml) was clearly distinguishable from plasma ECP (mean 1.0 ng/ml) by a factor of 8 (range: 5.6–11.6). The ECP released during clotting was markedly increased in the atopic dermatitis group (serum: plasma ratio 13.5, $p < 0.003$) compared with the other groups (6.7 and 5.6). EPX, having a higher plasma level, demonstrated a less pronounced release (serum: plasma ratios 2.0, 1.7 and 1.4), with no statistical difference between donor groups. Considering all donors together the levels of ECP and EPX in plasma and in serum were correlated to the number of eosinophils (coefficients of correlation 0.54–0.58, $p < 0.002$).**

**Key words:** Allergy, Atopic dermatitis, Eosinophil, Eosinophil cationic protein (ECP), Eosinophil protein X (EPX), IgE

### Introduction

Increased levels of plasma IgE and accumulation of eosinophils in blood and inflamed tissues are considered to be two important immunological features in allergic disease.[1,2] In animal models the interleukins 4 and 5 (IL-4 and IL-5) have been demonstrated to induce IgE synthesis, eosinophilia and eosinophil activation,[3–5] and these properties have been confirmed in human *in vitro* studies.[6–8] Thus, a link seems to exist between the high levels of serum IgE and eosinophil activation. To investigate whether patients with atopic dermatitis (severely increased IgE), inhalant allergy (moderately increased IgE) or normal individuals have different *in vivo* stimulation of eosinophils or have an increased susceptibility to stimuli, the mediator release from eosinophils was investigated. Moreover, this mediator release was compared with the donors' potential for *in vitro* production of IgE, which had previously been determined.

Many preformed and *de novo* synthesized mediators may be released from the eosinophil[2] and different experimental models have been suggested for monitoring mediator release.[8–10] The present approach was based on the fact that coagulation of blood induces a release of both eosinophil cationic protein (ECP) and eosinophil protein EPX (EPX) from the cells.[11] This model was chosen because it was speculated that this weak stimulus may only induce degranulation of *in vivo* activated eosinophils.

### Materials and Methods

*Allergic and non-allergic donors:* For the longitudinal study of the biological variation of the levels of ECP and EPX in serum and plasma six non-allergic, male donors were used.

Three groups of donors were admitted to the second part of the study. Group 1 showed atopic dermatitis with hyper-serum-IgE, and consisted of eleven individuals with serum IgE > 5 000 KIU/l. All patients in this group displayed atopic dermatitis according to the criteria of Hanifin and Rajka.[12] Group 2 consisted of ten allergic patients each with total IgE in the range 200–2 000 KIU/l, and inhalant allergy to pollen, house dust mites or animal dander. The diagnosis was established by case history, positive skin prick test and presence of allergen specific IgE. Group 3 consisted of ten non-allergic subjects, each with total IgE < 100 KIU/ml and no history of allergy.

The age profiles were similar in the three groups (35.8 ± 11.0, 39.0 ± 11.3 and 35.2 ± 5.6 years) as were the distributions between the two sexes: F/M, 7/4,

© 1994 Rapid Communications of Oxford Ltd

3/7 and 6/4. Total serum IgE in the three groups were: 12000 ± 9200, 485 ± 416, and 32 ± 30 KIU/l. Apart from the eosinophil counts ($0.51 \times 10^9$/l, $0.18 \times 10^9$/l and $0.13 \times 10^9$/l) no abnormal or varying haematological parameters were observed.

None of the donors had received systemic glucocorticoid treatment or cytostatic treatment 8 weeks prior to the studies, and no patients were undergoing allergen specific immunotherapy.

*Blood sampling:* Plasma and serum for ECP and EPX determinations were prepared from blood samples drawn in Vacutainers (Labco Inc., Bucks, UK) with EDTA or without anticoagulant, and allowed to clot for exactly 2 h at room temperature, which averaged at 21°C (range 20–23°C) during the study period. Counting of eosinophils was performed automatically using a Coulter counter.

*ELISA for ECP and EPX:* Serum and plasma levels of ECP and EPX were determined by ELISA methods described in detail elsewhere.[13,14] Briefly, both assays are sandwich type employing specific rabbit antibodies to ECP/EPX and the biotin–avidin amplification system. ECP and EPX are determined in the ranges of 15–1 000 and 60–2 000 pg/ml, respectively. Before measurement the samples were diluted in PBS (pH 7.4) containing 0.1% Tween 20, 0.1% CTAB (*N*-cetyl-*N,N,N*-trimethyl ammonium bromide), 20 mM EDTA, 0.2% human serum albumin.

In vitro *IgE synthesis:* The spontaneous and interleukin 4 (IL-4) induced IgE synthesis of peripheral blood mononuclear cells (PBMC) have previously been determined (Poulsen *et al.*, in preparation). Briefly, cells were isolated from heparinized blood using LymphoPrep™ (Nycomed, Oslo, Norway) and subsequently washed three times in medium. For *in vitro* synthesis of IgE, cells were grown in 96-well plates (200 µl at $2 \times 10^6$/ml) in the medium described by Yssel[15] at 37°C, in a humidified atmosphere containing 5% $CO_2$. Cells and medium were transferred to new wells at day 2, and the supernatants were harvested at day 11. Control cultures were acid extracted at day 0 as described,[16] and IgE was determined by an immunoradiometric assay (sensitivity 50 pg/ml).[17]

*Statistics:* The Mann–Whitney test for unpaired and the Wilcoxon test for paired observations were applied for testing differences between groups. Coefficient of correlation was calculated as Spearman's rho.

### Results

*Day-to-day variations in plasma and serum ECP in non-allergic donors:* The levels of ECP and EPX in serum were strongly dependent on the time and temperature during clotting of the blood sample. To investigate the biological variations when these two factors were carefully controlled, an experiment was performed in which the levels of serum and plasma ECP were determined in blood sampled from non-allergic donors during a period of 11 weeks. Table 1 illustrates the variation of ECP when determined in serum (coagulation time, 2.0 h) and EDTA-plasma of eight blood samples drawn from six non-allergic donors with intervals of 1 week or more. The mean serum level for this group was 8.07 ng/ml compared with 1.04 ng/ml for plasma. The coefficients of variation were larger for serum than for plasma values with averages of 77% and 49% respectively. In spite of these variations there was a clearly increased level of ECP in serum compared with plasma indicating an *in vitro* release of eosinophil proteins during coagulation of blood. Statistical analysis of the correlations between the number of eosinophils and the serum or plasma levels of ECP for each of the six donors did not reveal any significant correlation, indicating that intra-personal fluctuations in ECP levels were caused by other factors than the absolute number of eosinophils.

**Table 1.** Variation of ECP when determined in serum (coagulation time, 2 h, temp, 21°C) and plasma of eight blood samples drawn from six non-allergic donors during a period of 11 weeks

| | | | | | | | |
|---|---|---|---|---|---|---|---|
| **Serum ECP (ng/ml)** | | | | | | | |
| Donor | 1 | 2 | 3 | 4 | 5 | 6 | Mean |
| Mean | 9.79 | 4.25 | 9.58 | 14.14 | 5.18 | 5.46 | 8.07 |
| S.D. | 10.27 | 2.00 | 14.77 | 11.21 | 1.87 | 2.48 | — |
| C.V.(%) | 104.9 | 47.1 | 150.0 | 79.3 | 36.1 | 45.4 | 77.1 |
| **Plasma ECP (ng/ml)** | | | | | | | |
| Donor | 1 | 2 | 3 | 4 | 5 | 6 | Mean |
| Mean | 1.49 | 0.63 | 1.07 | 1.22 | 0.92 | 0.89 | 1.04 |
| S.D. | 1.14 | 0.24 | 0.58 | 0.60 | 0.35 | 0.36 | — |
| C.V.(%) | 76.5 | 38.1 | 54.2 | 49.2 | 38.0 | 40.4 | 49.4 |

Blood was drawn (cf. Materials and Methods) each week in weeks No. 1, 2, 3, 4, 5, 6, 7 and 11. No systematic changes were seen between the individual weeks. S.D. = standard deviation. C.V. = coefficient of variation.

*ECP and EPX in serum and plasma of non-allergic and atopic donors:* Fig. 1A shows the levels of ECP in plasma and serum from the three donor groups. As for the normal donors a marked higher level of ECP was seen in serum compared with plasma. This was the case for all three groups, but most pronounced in the atopic dermatitis group, which had an increased ($p < 0.003$) median serum-to-plasma ratio of 13.5 compared with 6.7 and 5.6 for the allergic and the non-allergic groups. The levels of EPX in serum and plasma generally demonstrated the same features as seen for ECP (Fig. 1B). The plasma level for EPX, however, was more than ten times that of ECP, making the *in vitro* release of EPX from the eosinophils into the serum less pronounced. Thus the median EPX serum-to-plasma ratios of the three

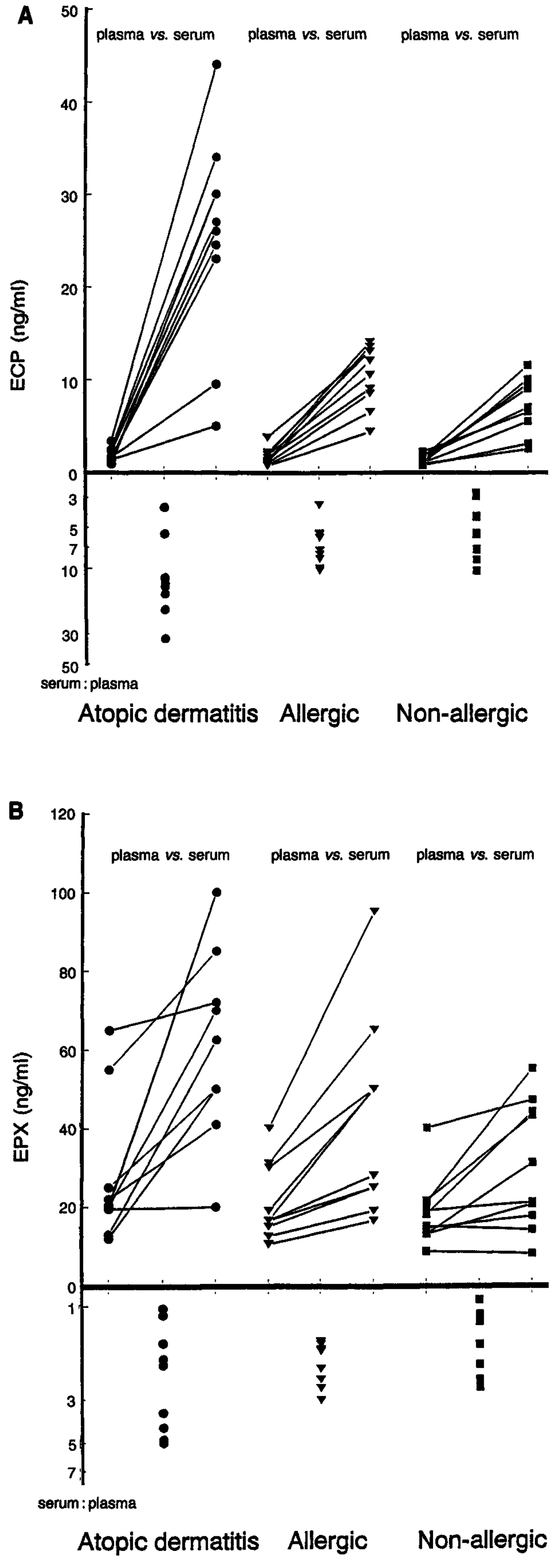

FIG. 1. Serum and plasma levels of ECP (A) and EPX (B). Blood samples were allowed to incubate for 2 h at 21°C and serum or plasma was harvested. The lower panel gives the ratios between the serum and the plasma levels for the individual donors.

groups were only 2.0, 1.7 and 1.4 respectively (not statistically different).

When considering all donors together, significant correlations were found between eosinophil number *vs.* serum and plasma levels of both ECP ($r = 0.58$ and $r = 0.54$, $p < 0.0001$ and $p < 0.002$) and EPX ($r = 0.55$ and $r = 0.58$, $p < 0.002$ and $p < 0.0001$). No such correlations were found within the three groups.

To investigate whether the higher serum-to-plasma ratio in the atopic dermatitis group reflected a higher state of activation (analogous to the 'releasability' concept for basophils[18]) or merely a higher number of eosinophils, the amounts of released protein were calculated on a per cell basis. Fig. 2 illustrates the amounts of released ECP and EPX (i.e., the serum value minus the plasma value) per eosinophil cell. There was a low but significant correlation ($r = 0.56$, $p < 0.001$) between the amount of the two proteins, and no differences were found among the three donor groups in respect of the distribution of ECP *vs.* EPX. However, the eosinophils generally seemed to release slightly more EPX than ECP. Moreover, the overall levels of released ECP and EPX varied more between individual donors than between groups.

*Correlation between IgE synthesis and release of eosinophilic proteins:* The atopic dermatitis group demonstrated high spontaneous and IL-4 induced IgE synthesis (Poulsen *et al.*, in preparation) and a higher number of eosinophils, including an increased release of ECP to serum during clotting of blood. To see if this correlation could be extended

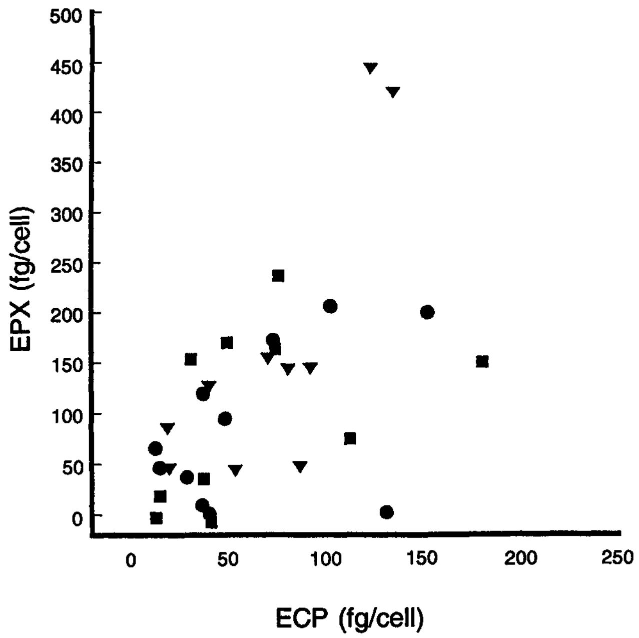

FIG. 2. The coagulation induced release of ECP and EPX per eosinophil cell. The cellular release was calculated as the difference between serum and plasma levels divided by the eosinophil cell number. Samples were incubated for 2 h at 21°C. ●, atopic dermatitis patients; ▼, allergic patients; ■, non-allergic patients.

**Table 2.** Correlation between the spontaneous and IL-4 induced IgE synthesis and eosinophil count, ECP level in serum, and the ECP serum : plasma ratio

| | Spontaneous IgE synthesis | | IL-4 induced IgE synthesis | |
|---|---|---|---|---|
| | *r* | *p* | *r* | *p* |
| Eosinophil count | 0.52 | 0.0076 | 0.46 | 0.0085 |
| ECP in serum | 0.53 | 0.0060 | 0.43 | 0.0168 |
| ECP serum : plasma ratio | 0.51 | 0.0099 | 0.40 | 0.0260 |

Spontaneous IgE synthesis was calculated as IgE in culture supernatants minus IgE in cultures at day 0. IL-4 induced IgE synthesis was calculated as the difference between supernatants ± IL-4.

to the entire material, i.e. the allergic and the non-allergic groups, the correlation was determined between the spontaneous and IL-4 induced synthesis on the one hand and the eosinophil parameters on the other. For EPX no correlation was found, whereas significant correlations were found between the spontaneous and the IL-4 induced IgE synthesis and eosinophil count, serum ECP and, consequently, the ECP serum : plasma ratio (Table 2). The correlation between ECP release and IgE synthesis was not superior to the IgE-synthesis-eosinophil count correlations, indicating no direct links between eosinophil 'releasability' and potential for IgE synthesis. Moreover, when ECP release was calculated on a 'per cell basis' as described above, no significant correlations were found for either spontaneous or IL-4 induced IgE synthesis. Generally, the eosinophil count, serum levels and serum : plasma ratios correlated better to the spontaneous synthesis, compared with the synthesis induced by IL-4.

### Discussion

Several studies have shown the strong correlation between eosinophilia and IgE related diseases such as allergy,[2] atopic dermatitis[19] and parasitosis,[20] but the exact mechanisms by which the eosinophils are recruited and activated are still enigmatic. As Th2 type cytokines like IL-5 and granulocyte macrophage colony stimulating factor (GM–CSF) have been incriminated in the degranulation,[5] it was speculated that eosinophil activation would be present in donors featuring an active IgE synthesis.

It has been suggested that serum levels of eosinophilic proteins may be used as markers of the number of eosinophils in peripheral blood. However, we have previously demonstrated a large discrepancy between serum and plasma levels, and a strong time and temperature dependent release of ECP and EPX from eosinophils when a blood sample is allowed to coagulate.[11] This indicated that the eosinophils are induced to release the proteins by the coagulation process. Moreover, these data suggested that measurements of serum ECP should be carried out very cautiously using standardized procedures. To test the day-to-day variations, a longitudinal study of non-allergic donors was performed, and as illustrated in Table 1, variations of 50–80% occurred between different days. The ECP levels in plasma could, however, be clearly discriminated from the corresponding serum values that were increased by a factor of 8. Accordingly, the second part of the study was carried out, in which the coagulation induced release of ECP and EPX was studied in donors of varying atopic status.

Regarding the ECP release a marked difference was observed between the atopic dermatitis group and the two other groups (Fig. 1). It cannot be concluded, however, that this difference stems from different levels of activation. More likely the difference reflects the fact that the release of ECP during the clotting of blood is related only to the number of eosinophils. This is emphasized in Fig. 2, in which no special release pattern of ECP or EPX on a per cell basis is seen in any of the three groups. As we have not determined the number of normodense *vs.* hypodense eosinophils in the blood of the patients, it is also possible that the blood eosinophils have not been activated and that activated eosinophils are located exclusively in the target organ of the inflammatory process.

From Table 2 it is seen that correlations exist between the serum level of ECP and the serum : plasma ratio of ECP on the one hand and the spontaneous and IL-4 induced IgE synthesis on the other. The two eosinophil parameters do not, however, correlate better than the eosinophil count, suggesting that the atopic status affects only the eosinophilopoiesis and not the activation degree of the eosinophils in the peripheral blood.

Several experimental models have been suggested for monitoring mediator release of the eosinophil granulocyte.[8–10] It should be emphasized that our approach, i.e. mediator release caused by coagulation of blood, induces a substantially lower release than the one induced by complement- or IgA/IgG-dependent mechanisms, which can induce even normodense, i.e. non-activated eosinophils, to degranulate.[8] The coagulation induced degranulation was chosen, however, because it was speculated that this weak stimulus may only induce degranulation of *in vivo* activated eosinophils. Although the exact mechanism by which the cells degranulate is not known, it seems to be a calcium-dependent secretion because heparinized plasma contains higher amounts of ECP and EPX.[11]

In conclusion we have found a correlation between the number of eosinophils and the release of ECP and EPX to serum during a specified period. Provided that the technical difficulties of standardizing drawing and coagulation of blood are solved, the release of eosinophilic proteins probably reflects a combination of the number of the activation state of the eosinophils. The group of inhalant allergic donors fell between the atopic dermatitis and the non-allergic individuals in both eosinophil numbers and protein levels, but no significant differences to the latter group could be demonstrated. During the eosinophilopoiesis the cell spends a relatively brief period in circulation before it is recruited to inflammatory foci. Thus it is possible that higher numbers of activated eosinophils and hence a larger coagulation induced release can be found in patients in a more active state of their disease.

### References

1. Ishizaka K, Ishizaka T. Identification of gamma-E-antibodies as a carrier of reaginic activity. *J Immunol* 1967; **99**: 1187–1198.
2. Wardlaw AJ, Kay AB. The role of the eosinophil in the pathogenesis of asthma. *Allergy* 1987; **42**: 321–335.
3. Coffman RL, Carty J. A T cell activity that enhances polyclonal IgE production and its inhibition by interferon γ. *J Immunol* 1986; **136**: 949–954.
4. Sanderson CJ. The biological role of interleukin 5. *Int J Cell Cloning* 1990; **Suppl 1**: 147–153.
5. Sanderson CJ, Campbell HD, Young IG. Molecular and cellular biology of eosinophil differentiation factor (interleukin-5) and its effects on human and mouse B cells. *Immunol Rev* 1988; **102**: 29–50.
6. Pene J, Rousset F, Briere F, *et al.* IgE production by normal human lymphocytes is induced by interleukin 4 and suppressed by interferon γ and α and prostaglandin E2. *Proc Natl Acad Sci USA* 1988; **85**: 6880–6884.
7. Pene J, Rousset F, Briere F, *et al.* Interleukin 5 enhances interleukin 4-induced IgE production in normal human B cells. The role of CD23 antigen. *Eur J Immunol* 1988; **18**: 929–935.
8. Fujisawa T, Abu-Ghazaleh R, Kita H, Sanderson CJ, Gleich GJ. Regulatory effect of cytokines on eosinophil degranulation. *J Immunol* 1990; **144**: 642–646.
9. Winqvist I, Olofsson T, Olsson I. Mechanisms for eosinophil degranulation; release of the eosinophil cationic protein. *Immunology* 1984; **51**: 1–8.
10. Carlson M, Håkansson L, Peterson C, Stålenheim G, Venge P. Secretion of granule proteins from eosinophils and neutrophils is increased in asthma. *J Allergy Clin Immunol* 1991; **87**: 27–33.
11. Reimert CM, Poulsen LK, Bindslev-Jensen C, Kharazmi A, Bendtzen K. Measurements of eosinophil cationic protein (ECP) and eosinophil protein X/eosinophil derived neurotoxin (EPX/EDN): need for standardized sample processing due to time and temperature dependent spontaneous release *in vitro*. *J Immunol Methods* 1993; **166**: 183–190.
12. Hanifin JM, Rajka G. Diagnostic features of atopic dermatitis. *Acta Derm Venereol Stockh* 1980; **92**: 44–47.
13. Reimert CM, Venge P, Kharazmi A, Bendtzen K. Detection of eosinophil cationic protein (ECP) by an enzyme-linked immunosorbent assay. *J Immunol Methods* 1991; **138**: 285.
14. Reimert CM, Minuva U, Kharazmi A, Bendtzen K. Eosinophil protein X/eosinophil derived neurotoxin (EPX/EDN): detection by enzyme-linked immunosorbent assay and purification from normal urine. *J Immunol Methods* 1991; **141**: 97.
15. Yssel H, de Vries JE, Koken M, van Blitterswijk W, Spits H. Serum-free medium for generation and propagation of functional human cytotoxic and helper T cell clones. *J Immunol Methods* 1984; **72**: 219–227.
16. Poulsen LK, Baron L, Heinig JH, Stahl Skov P, Bendtzen K. Biomolecular regulation of the IgE immune response. I. Cell-associated IgE and *in vitro* IgE synthesis. *Allergy* 1992; **47**: 560–567.
17. Poulsen LK, Malling H-J, Søndergaard I, Weeke B. A sensitive and reproducible method for the determination of subnanogram quantities of immunoglobulin E (IgE). *J Immunol Methods* 1986; **92**: 131–136.
18. Lichtenstein LM, MacGlashan DW. The concept of basophil releasability. *J Allergy Clin Immunol* 1986; **77**: 291–294.
19. Kapp A. The role of eosinophils in the pathogenesis of atopic dermatitis—eosinophil granule proteins as markers of disease activity. *Allergy* 1993; **48**: 1–5.
20. Finkelman FD, Pearce EJ, Urban JFJ, Sher A. Regulation and biological function of helminth-induced cytokine responses. *Immunol Today* 1991; **12**: A62–A66.

ACKNOWLEDGEMENTS. The excellent technical assistance of Lena Baron and Ulla Minuva and the secretarial assistance of Anne Larsen are gratefully acknowledged. This study was supported by the Biotechnological Center for Signalling Peptides.

**Received 24 February 1994; accepted in revised form 28 March 1994**
